# Toxicological Effects of Inhaled Crude Oil Vapor

**DOI:** 10.1007/s40572-024-00429-8

**Published:** 2024-01-24

**Authors:** Jeffrey S. Fedan, Janet A. Thompson, Tina M. Sager, Jenny R. Roberts, Pius Joseph, Kristine Krajnak, Hong Kan, Krishnan Sriram, Lisa M. Weatherly, Stacey E. Anderson

**Affiliations:** Health Effects Laboratory Division, National Institute for Occupational Safety and Health, 1095 Willowdale Road, Morgantown, WV 26505 USA

**Keywords:** Crude oil, Crude oil vapor, Inhalation, Hazard identification

## Abstract

**Purpose of Review:**

The purpose of this review is to assess the toxicological consequences of crude oil vapor (COV) exposure in the workplace through evaluation of the most current epidemiologic and laboratory-based studies in the literature.

**Recent Findings:**

Crude oil is a naturally occuring mixture of hydrocarbon deposits, inorganic and organic chemical compounds. Workers engaged in upstream processes of oil extraction are exposed to a number of risks and hazards, including getting crude oil on their skin or inhaling crude oil vapor. There have been several reports of workers who died as a result of inhalation of high levels of COV released upon opening thief hatches atop oil storage tanks. Although many investigations into the toxicity of specific hydrocarbons following inhalation during downstream oil processing have been conducted, there is a paucity of information on the potential toxicity of COV exposure itself.

**Summary:**

This review assesses current knowledge of the toxicological consequences of exposures to COV in the workplace.

## Introduction

There were approximately 186,000 workers employed in August 2020 in the USA in the oil and gas extraction industry [1–4]. At several points in the production chain from the ground to the refinery, including drilling, storage, pipelines, and cleaning operation, workers are potentially exposed by inhalation to crude oil vapor (COV) or by getting crude oil on their skin [5–11]. Crude oil is a complex mixture of organic chemicals, metals, salts, and semivolatile species [1, 12]. The relative concentrations of the various components depend on the drilling fields, depth of drilling, and geographical location of extraction [1]. The rate of illness and injury in the upstream segment of the oil and gas extraction industry, which includes exploration, drilling, storage, and transportation of crude oil to refineries, is comparable to that of workers in all industries [13], but the rate of fatality is higher [14], and many physical hazards have been recognized. However, the adverse health effects of crude oil exposure in upstream workers are less clearly defined. There is abundant information regarding the short- and long-term health effects resulting in humans from exposure to crude oil *components*, but the majority of information on the health effects of raw COV exposure comes from its effects on cleanup workers after oil spills [15–17]. The purpose of this review is to assess the toxicological consequences of COV exposure in the workplace through evaluation of the most current epidemiologic and laboratory-based studies in the literature.

## Epidemiologic Studies in Crude Oil–exposed Workers

Workers in the oil and gas industry are exposed to hydrocarbon vapors (HCV) and volatile organic compounds (VOCs) while conducting routine tasks in or around petroleum storage tanks, such as manual gauging, sample collection, flow back operations, and tank cleaning and maintenance [18–21]. Opening of the thief hatch atop petroleum storage tanks results in the release of HCV/VOC plumes (> 100,000 ppm), which can result in worker exposure at or downwind from the tank [21–25]. From January 2010 to March 2015, nine oil worker deaths were attributed to a combination of oxygen deficiency and HCV/VOC inhalation following opening of thief hatches [26], with exposure to hydrogen sulfide being ruled out as a cause of death [27].

In a series of retrospective, cross-sectional follow-up analyses of petroleum company records, the overall rate of mortality of workers in all job categories was essentially comparable to that of the US population, although increases were observed for certain diseases (cancers and blood diseases) [28–33]. Cancer incidence (acute myelogenous leukemia and multiple myeloma) was correlated to historical crude oil exposure in one study [34]. In addition, morbidity studies of petroleum workers noted respiratory and heart diseases among the five most prevalent illnesses [35–37]. These studies did not consider the healthy worker effect (the notion that the health of workers is generally better than that of the overall population [38]), other comorbidities in their study designs, or upstream stream petroleum workers in their studies. Limited worker exposure data exists for the petroleum industry [39]. Because it can take 10 years or longer for coronary, respiratory, and other chronic disease to develop following occupational exposure, these studies were not able to directly link crude oil to the development of these diseases.

Important insights into the potential adverse health effects of inhaled COV on upstream petroleum workers are provided from the early and chronic symptoms occurring after oil tanker accidents, approximately 38 of which have occurred over the past few decades [39]. Because the petroleum product carried in each tanker varies from ship to ship, it is difficult to associate a particular set of symptoms with a specific type of oil. Adverse health effects in oil spill cleanup and recovery response workers were not investigated after every incident. Most often, the observed effects followed inhalation exposure to weathered petroleum, which lacks many volatile constituents. Many of the investigations involved short-term cross-sectional studies without obtaining biological samples (e.g., blood) or physiological measurements for analysis. Despite these caveats, early and follow-up investigations of cleanup workers and residents in proximity indicated that exposures were associated with a variety of acute, sub-chronic, and chronic symptoms including upper and lower respiratory, neurological (headache, nausea, vomiting, dizziness), liver function, hematological function, and ocular abnormalities [40–75]. Other outcomes, i.e., genotoxic [75–77], reproductive [78, 79], endocrine [80], and effects of skin exposure [81], have been reported in workers. Some acute effects of oil exposure appear to be reversible, such as lung function decrements in Deepwater Horizon cleanup workers [82].

It is of importance to note that chemical dispersants are employed to minimize damage to the environment during oil spill cleanup, and workers are potentially exposed. The bulk of the studies of the biological effects of dispersants address effects in non-mammalian species; investigations in mammals are limited in number (see, for example, [83]). In rodent models, inhalation exposure to COREXIT® EC9500A caused myriad toxicological responses in skin, the cardiovascular system, lungs, and brain [84–88]. Cleanup workers are potentially exposed to a mixed exposure consisting of weathered oil vapor and dispersant to varying degrees, each with its own toxicological profile. There exists a knowledge gap in the understanding of additive or possible synergistic interactions of oil vapor and dispersant exposure in crude oil cleanup workers.

Chronic exposure to crude oil over periods of years may lead to unrecognized morbidities. Follow-up studies indicate that persistent respiratory symptoms, markers of lung injury, and chromosome damage persisted in workers for at least 2 years following cleanup of bunker oil (heavy oil) from the 2002 *Prestige* spill [44, 52]. Decrements in lung function were observed after the *Tasman Spirit* (Iranian light crude) shipwreck [82]. Investigations into the long-term toxicological effects associated with cleanup of oil spills have been conducted, and some symptoms appear to have waned over time (years), for example, the respiratory symptoms [62, 63, 80]. On the other hand, elevated heart attack risk [65], reduced lung function in some cleanup workers [64], and genetic alterations [67] persisted for several years.

Studies performed to examine the long-term effects of crude oil exposure indicated that workers maintaining tanks in ships (jobs including inspection, cleaning, repair) experienced immune suppression characterized by decreases in IgM and IgA levels (Kirkeleit et al*.*, 2006). Oil shale workers experience respiratory, neurological, cardiovascular, and other physical problems [81], and, in crude oil workers in the USSR, hypertension was observed [82]. Subsequently, cardiovascular morbidity was reported in a study of upstream oil workers [89]. Decrements in respiratory function are known to exist 1–3 years after acute inhalation exposure to aromatic hydrocarbons, which are also present in crude oil vapor (e.g., benzene, [90, 91]).

### Hepatic Effects

The liver is an organ critical for biotransformation of xenobiotic agents, including hydrocarbons and other volatile components of crude oil [92], for removal from the body. Human exposure to crude oil is associated with changes in the liver and its function. Sonographic assessments of petroleum-induced hepatotoxicity among oil workers revealed enhanced echogenicity, fatty liver, and increased liver size [93, 94]. Assessment of liver function indices among volunteers participating in the Deepwater Horizon (DWH) oil spill cleanup operations showed increased liver marker enzymes, aspartate transaminase (AST) and alanine transaminase (ALT), which persisted in follow-up studies conducted 7 years after the initial assessment [56–58]. These long-term effects resulting from oil spill exposure suggest that oil spill response workers may be at risk for developing hepatotoxicity and altered liver function [56, 58, 93, 94].

## Potential Toxicologic Mechanisms of Downstream Petroleum Products

Several investigations have examined the effects of upstream and downstream petroleum products in non-human mammalian systems or non-mammalian systems to define toxicity and potential mechanisms. Upstream production includes extraction and production, while downstream production includes post-production of crude oil activities at refineries, petrochemical plants, and storage tanks. The many adverse biological effects of downstream petroleum products [95, 96] are not summarized here, but information has been reported in the literature and made available by the petroleum industry [97–99]. Their relevance to modeling inhaled COV adverse health effects in humans is limited. A few inhalation studies of the effects of downstream petroleum products have been conducted [100–102], but we discovered only one [103] involving crude oil. Other identified studies rely, instead, on oral, dermal, or i.p. administration or the use of in vitro cells or cell lines. Table 1 summarizes the non-carcinogenic end points and cellular pathways induced by crude oil exposure in mammalian and in vitro systems.

**Table 1:** Summary of non-carcinogenic end points and cellular pathways induced by crude oil exposure in mammalian and in vitro systems

Endpoint	Major Findings	Exposure	Species or system	Reference
GENETIC Genotoxicity	• DNA damage in peripheral blood leukocytes	Inhalation	Rat	[101]
	• Increased expression of nucleotide excision repair (NER) pathway genes	In vitro and immersion	*C. elegans* and zebrafish	[103]
Gene expression Gene expression	• Interleukin-18 (IL-18), IL-10, inducible nitric oxide synthase (iNOS), cyclooxygenase 2 (COX-2), complement cytolysis inhibitor (CLI) and thyroid hormone receptor (THR) down-regulated • IL-2 and heat shock protein 70 (HSP70) up-regulated	Dietary	Mink	[102]
	• aryl hydrocarbon receptor (AhR) interactions	In vitro	Rat hepatoma cells	[127]
LIVER Function/morphology	• Elevated liver enzymes (AST,ALP, ALT), hyperbilirubinemia, excessive triglyceride accumulation in hepatocytes	Ingestion	Rabbit	[128]
Enzyme induction	• Induction of CYP 1A2, COX2, and 5-LOX • Induction of leukotriene B4 (LTB(4)	In vitro	Human cell lines	[129]
	• Induction of liver mitochondrial DNA synthesis	Intraperitoneal (i.p) injection	Guinea pig	[130]
	• Induction and increased activity of hepatic cytochrome P-450 and xenobiotic-metabolizing enzymes	Gavage	Rat Mouse	[131, 132]
	• Increased liver weight, hepatic total proteins, RNA, glycogen, cholesterol, triglycerides,	Gavage	Mouse	[133]
	• Reduction in liver mitochondrial and microsomal ATP-dependent calcium uptake activity	Gavage	Mouse	[114]
	• Inhibition of mitochondrial respiration and total lipids	Gavage	Mouse	[134]
	• Increased bile production and DNA adduct formation, reduction of lactate metabolism	Liver perfusion	Rat	[135]
LUNG	• Increased malondialdehyde (MDA); decreased superoxide dismutase (SOD) and catalase (CAT) activities, glutathione (GSH) level	Inhalation	Rat	[136]
	• Increased lipid peroxidation, loss of cell respiration in pulmonary alveolar macrophage	In vitro (PAM)	Rabbit Rat	[137]
CARDIOVASCULAR	• Increased systolic and diastolic blood pressure, mean arterial pressure (MAP), heart rate (HR) and baroreflex sensitivity	Inhalation	Rat	[122]
	• Increased heart rate, reduced diastolic pressure, increased heart weight	Inhalation	Rat	[138]
SKIN	• Increased UV sensitivity	In vitro	Rat	[139]
	• Reduced epidermal IA and Thy-1 expression,	In vitro	Rat	[140]
GASTROINTESTINAL	• Altered enzyme activity and intestinal flora	Gavage	Rat	[141]
ENDOCRINE	• Estrogen (alpha and beta) and androgen receptor-mediated responses in yeast assays	In vitro	Yeast	[142]
	• Induced cell proliferation and mRNA expression estrogen-dependent protein pS2	In vitro	Human cell line	[143]
	• Anti-estrogenic activity, antagonistic binding to estrogen receptors	In vitro	Human cell line	[144]
	• elevated generation of reactive oxygen species (ROS)	In vitro	Human cell line	[145]
REPRODUCTIVE and DEVELOPMENT	• Fetal utero death, decreased body weight	Dermal	Rat	[146]
	• Kits of exposed mothers had decreased survival, reduced reproductive success	Dietary	Mink	[147]
	• Decrease in embryonic survival and rhodopsin mRNA expression • Increased overall development deformities	Immersion	Zebra fish	[148]
HEMATOLOGY	• Hematological changes, anemia, altered white blood cell numbers, lymph node and splenic atrophy, genotoxicity in immune cells	Gavage	Human	[149]
	• Modulation of cytokine gene expression, increased susceptibility to infectious diseases Increased erythrocyte malate dehydrogenase (MDH) and lactate dehydrogenase (LDH), glucose-6-phosphate dehydrogenase (G-6-PDH), and catalase activity			[150]
	• Inhibit platelet aggregation and Ca^+^ uptake	In vitro	Rat	[151]
	• Altered blood cell profile. increased packed cell volume (PCV), granular leucocytes; reduced lymphocytes and monocytes	Dietary	Goat	[152]
	• Decreased packed cell volume (PCV) • Increased hemoglobin	Oral	Mouse	[153]
ORGAN WEIGHT	• Liver enlargement, thymus, and spleen atrophy	Dermal	Rat	[154]

A comprehensive review of the toxicology of oil in vertebrates is provided by Takeshita et al*.* [75]. Inhalation of a heavy distillate of coal liquid increased heart rate and blood pressure in response to isoproterenol [104], a β-adrenoceptor agonist. While the composition of coal distillate is very different from crude oil, both crude oil and coal distillate contain complex and aromatic hydrocarbon compounds, and therefore, the effects of distillate exposure could be predictive of COV’s adverse effects. Attempts have been made using animal organ/tissue end-points, none of them involving pathophysiological measurements, to predict the acute and repeated dose toxicity of petroleum mixtures in humans based upon knowledge of certain well-characterized PAH components [97, 105–107].

## Pathophysiological Effects of COV Exposure in Animal Models

Until recently, comprehensive studies had not been undertaken to investigate the potential pathophysiological effects of COV inhalation on the respiratory, cardiovascular, central nervous, and immune systems and kidneys using mammalian animal models, although one investigation using rats and mice examined the pulmonary effects of a heavy fuel oil blend resembling the oil spilled from the *Prestige* oil tanker [108]. A great deal of information is known regarding the toxicities of the individual components found in crude oil [90, 91, 109, 110], but the toxicity of COV exposure, as occurs in workers, is not well understood.

Recently, investigations [92, 111–118] were conducted to evaluate the toxicity of inhaled COV generated from *Deep Water Horizon* (DWH) surrogate crude oil in a rat model to gain a greater understanding of the adverse health effects of COV inhalation in upstream petroleum workers [49, 74, 119]. The DWH surrogate crude oil provided by British Petroleum (BP) Exploration and Production, Inc., similar to the oil associated with the 2010 Deepwater Horizon spill from the Macondo Well in Mississippi Canyon Block 252, was characterized in McKinney et al. [95]. In vivo and in vitro endpoint measures were made 1 and 28 days after an acute, whole-body exposure to 300 ppm COV for 6 h, or 1, 28, and 90 days after a sub-chronic exposure (300 ppm VOCs for 6 h/days × 4 days/week × 4 weeks). The COV level used in the animal exposures brackets those that have been measured at thief hatches and flowback operations in the field.

### Pulmonary Effects

Studies in the lung [114] indicated that no changes in respiratory system resistance, elastance, tissue damping, tissue elastance, Newtonian resistance, hysteresivity, or reactivity to inhaled methacholine (MCh) were elicited by COV inhalation. Ninety days after sub-chronic exposure to COV, the inhibitory effect of the airway epithelium on in vitro reactivity of airway smooth muscle to MCh was enhanced. Cholinergically mediated, neurogenic contractile responses of airways in vitro were unaffected following COV exposure. Investigation of ion transport by the tracheal epithelium revealed that neither basal resistance nor short-circuit current was modified by COV 28- and 90-day post-exposure, but Cl¯ and Na^+^ transport were enhanced 1 day post-exposure. No significant effects in lung vascular permeability were observed at any end point time following acute or sub-chronic inhalation of COV. No histological exposure-related morphologic alterations were observed in the lungs of the COV-exposed rats. Evidence of an inflammatory response was obtained from analysis of bronchioalveolar lavage fluid (BALF). There were no significant effects of COV on BALF lactate dehydrogenase activity, a common measure of cytotoxicity. At 1 day post-exposure to COV, there were significant increases in leptin, interleukins (IL)-1β and IL-10, IFN-γ-inducible protein (IP-10), and lipopolysaccharide-induced CXC chemokine (LIX), and, at 28 days, vascular endothelial growth factor (VEGF) was significantly increased. At 90-day post-exposure, COV exposure caused decreases in granulocyte–macrophage colony stimulating factor, interferon-γ, and neutrophil activating protein CXCL1. Examination of a large panel of cytokine mediators revealed no changes in their levels in BALF following COV inhalation. No changes in the total cells, alveolar macrophages, or neutrophils occurred at any of the post-exposure measurement periods. Oxidant production by macrophages was minimally affected. No significant changes in the global gene expression profiles were detected in the lungs of the acute COV-exposed animals at 1 day post-exposure. Significant differential expression of 47 genes and 52 genes was detected in the acute (300 ppm VOCs × 6 h/day × 1 day) 1 and 28 days and sub-chronic (300 ppm VOCs × 6 h/days × 4 days/week × 4 weeks) COV-exposed rat lungs at 28 days post-exposure, respectively. The changes in the lung gene expression profile detected in the absence of any detectable lung toxicity in the COV-exposed rat lungs were likely due to the greater sensitivity of the transcriptome to respond compared to pathophysiological, histological, and biochemical endpoints of target organ toxicity.

### Cardiovascular and Renal Effects

Cardiovascular and renal effects of COV exposure were examined [115]. Acute COV exposure decreased left ventricular end-systolic pressure 1 day post-exposure and left ventricular end-diastolic pressure 28 days post-exposure. Dobutamine-induced vasoconstriction was not markedly affected 1 day following exposure, but was reduced 28 days following exposure. Diastolic and mean arterial pressures also were reduced 1 day following acute COV treatment. The acute exposure resulted in an increase in oxidative stress in COV-exposed animals 24 h after exposure, but by 28 days following exposure measures of oxidative stress was reduced in both groups of animals. Analysis in heart and kidney revealed a myriad of COV-induced, post-exposure changes in transcript levels of multiple inflammatory cytokines and enzymes at varying post-exposure time points: IL-6, hypoxia-induced factor-1α, TNF-α, inducible NO synthases, endothelial nitric oxide synthase, tumor necrosis factor, IL-1β, Il-6, tissue inhibitor of metalloproteinase, catalase, and superoxide dismutase. Protein markers that have been associated with the presence of cardiovascular or renal dysfunction also were measured after acute COV exposure. There were no significant exposure-related differences in concentrations of any of the proteins in the heart or kidneys.

### Neurological Effects

Sriram et al. [117] investigated the effects of COV inhalation on several aspects of nervous system function. As alkanes, cycloalkanes/naphthenes, and aromatic hydrocarbons present in COV are membrane-perturbing compounds, it was hypothesized that they can affect neuronal membranes causing aberrant synaptic signaling and impaired neurotransmission, defects which can ultimately culminate in neural damage. Indeed, acute or sub-chronic exposure to COV was shown to cause brain region- and time-specific alterations in the levels of the biogenic neurotransmitters, norepinephrine (NE), epinephrine (EPI), dopamine (DA), and serotonin (5-hydroxytryptamine (5-HT) in the olfactory bulb (OB), striatum (STR), and/or midbrain (MB). Monoamines like DA, NE, EPI, and 5-HT are known to modulate olfactory function, regulate odor inputs, and play a role in depression and anxiety disorders. Acute or sub-chronic COV exposure altered the expression of several synaptic proteins including synaptophysin 1 (SYP), synaptotagmin (SYT), and/or tyrosine 3-monooxygenase/tryptophan 5-monooxygenase activation protein epsilon (YWHAE) in the brain. Specifically, sub-chronic COV exposure induced SYP and SYT protein expression in all brain regions examined. SYP and SYT are involved in synaptic transmission, and their increased expression is associated with long-term potentiation of synaptic mechanisms for altering neurotransmitter release and olfactory memory formation. Abnormal accumulation of synaptic proteins might result in modulation of neurotransmission and functional disturbances. SYP, a major synaptic vesicle protein, is also involved in vesicle sorting, priming, synaptic biogenesis, synapse formation, exocytosis, and endocytosis. It plays a key role in axonal neurotransmitter release, including release of DA. Dynamic changes in the expression of presynaptic and axonal proteins are known to precede dopaminergic neurotoxicity. Indeed, changes in the expression of dopaminergic and PD-related proteins, tyrosine hydroxylase (TH), Parkinson disease-5, autosomal dominant (PARK5; also known as ubiquitin carboxy-terminal hydrolase L1/UCHL1), and Parkinson disease 7, autosomal recessive early-onset (PARK7; also known as Parkinsonism-associated deglycase or oncogene DJ1) were also seen after COV exposure. Acute exposure to COV increased TH and PARK7 proteins in the dopaminergic brain areas, STR and MB. Sub-chronic COV exposure upregulated PARK5 and PARK7 proteins in the STR and MB. TH is the rate-limiting enzyme in the synthesis of DA. Modulation of its function or loss of TH protein is an index of dopaminergic injury. PARK genes are normally involved in affording neuroprotection against oxidative stress resulting from mitochondrial dysfunction. In humans, loss-of-function mutations in PARK genes are associated with early-onset Parkinsonism [118]. The increased expression of PARK5 and PARK7 in the STR and MB following COV exposure reflects a potential protective mechanism to clear over-expressed or defective/abnormal proteins, control oxidant damage, and maintain mitochondrial integrity.

### Immunological Effects

The immunotoxicity of COV following acute and sub-chronic inhalation exposure was investigated [119]. The immune cell subsets present in lung lymph node (LLN), bronchoalveolar lavage, and spleen, along with total cellularity, were characterized. Acute COV inhalation exposure increased BALF cellularity, CD4 + and CD8 + cells, and absolute and percent CDllb + cells only at 1 day post-exposure. Sub-chronic inhalation exposure resulted in a decreased frequency of CD4 + T-cells at 1 day post-exposure and an increased number and frequency of B-cells at 28 days post-exposure in the LLN. A significant elevation in the number and frequency of B-cells was observed in the spleen at 1 day post-exposure. Exposure to COV, acutely and sub-chronically, suppressed NK cell function 1 d post exposure. No changes were observed at the other post exposure time points. Serum chemistries and complete blood counts were analyzed following COV exposure. No consistent hematological changes were observed at all the time points evaluated. The IgM response to SRBC was examined to evaluate whether exposure to COV was immunosuppressive. No significant change in the PFC/spleen or specific (PFC/10^6^ cells) IgM antibody activity against SRBC was observed at any of the post-exposure time points.

### Hepatic Effects

In the scientific literature, there are limited studies describing potential mechanisms involved in crude oil exposure–induced effects in the liver. Exposure of mice to the Prudhoe Bay crude oil resulted in an increase in liver weight, increased hepatic total protein, and increased total lipids [120]. Rats exposed to Nigerian bonny light crude oil (BLCO) exhibited hepatic degeneration, which was associated with reduced γ-glutamyl transferase activity and elevated levels of serum aminotransferases, glutathione, hydrogen peroxide, and malondialdehyde, suggesting oxidative stress [121]. BLCO has also been shown to inhibit calcium influx and induce mitochondrial DNA, leading to mitochondrial swelling and dysfunction in the liver of guinea pigs [122]. The various components of crude oil, e.g., BTEX and others, however, have been examined extensively for their effects on the liver [123–126].

## Conclusions

The broadest conclusion from the studies with DWH surrogate crude oil was the lack of biological effects in several organ systems apart from the nervous and immune systems. Where changes were evident, the brain and immune systems appeared to be the most responsive. Surprisingly, measurement of diverse in vivo and in vitro markers of ventilatory and non-ventilatory lung function revealed that the lungs were refractory to the effects of COV administered acutely or sub-chronically, despite being a portal of entry of the vapor. COV exposure resulted in changes in the levels of several markers in the brain and kidney. These did not follow a strict post-exposure continuum time-dependence, nor were they relatable to acute *vs.* sub-chronic exposure paradigms. For the most part, COV-induced changes were overcome by 28–90 days post-exposure.

The profile of immune cell subsets in BALF at 1 day post-exposure revealed COV-induced alterations; other immune profiles were not affected. Whereas it can be safely assumed that inhaled COV triggered responses in other organ systems mostly after having entered the lungs, whole-body exposure of the animals to the vapor exposed their skin directly. Future studies using nose-only exposures to COV will be needed to ascertain whether responses comparable to those observed in the current investigation were triggered or influenced by a dermal exposure component, as could potentially be experienced in upstream oil workers.

The question of whether the findings made here are directly relevant to the adverse health effects observed in the DWH or other oil-spill cleanup workers can be raised. Here, it is difficult to draw definitive conclusions. First, the spilled oil to which the workers were exposed was weathered by the environment and, over time, its composition was changed. Second, chemical dispersants were employed to minimize damage to the environment and workers were potentially exposed to these in addition to weathered oil vapor. Oil spill cleanup workers experience mixed exposures consisting of weathered oil vapor and dispersant to varying degrees, whereas exposures in upstream oil workers consist of neat crude oil and its vapor. The findings made in each context cannot be extrapolated across exposure scenarios.

There is limited information about effects of COV at the molecular/cellular level in mammalian biological systems. Possible mechanisms, derived primarily in non-mammalian species include induction of hepatic enzymes and leukotriene formation, enzyme release from AMs, anti-estrogenic activity and ROS, altered gene expression, increased expression of nucleotide excision repair pathway genes, products of xenobiotic metabolism, DNA adduct formation, differential expression of miRNA, altered mitochondrial function, DNA damage, chromatin changes, aryl hydrocarbon receptor interaction, androgen and estrogen receptor interaction, oxidation/peroxidation systems, and decrease in rhodopsin mRNA expression and increase in hematopoietic regulator, runx1 (runt-related transcription factor 1). For example, in the heart, the decreases in ROS levels may have been involved in decrements of function, i.e., blood pressure changes. However, the various changes in airway epithelial ion transport are difficult to ascribe mechanistically to any of the biomarkers mentioned above; for example, there are no reports of the aryl hydrocarbon receptor as a component of the pathways regulating airway epithelial Na^+^ or Cl¯ channels. It remains for future studies to define the transduction pathways affected by COV in the organ systems that have been studied and those that have not, e.g., liver.

The way COV (inhaled or dermal) interacted with organs in the rat to alter function is unknown. Was COV taken up by the lungs (and skin) and distributed subsequently throughout the body, or were signals sent from the lung to other organs via the blood?

Much work remains to characterize further the toxicity of inhaled COV, i.e., its effects after longer exposure periods and over a range of exposure concentrations, and, accompanying that, its residual effects during longer post-exposure periods. Inasmuch as workers at oil well fracking sites are exposed to varying climate conditions, such that an exposure to a constant level of COV in the ambient air is not achieved, models that recapitulate cyclic/periodic exposures to the vapor could better estimate workplace exposure hazards to itinerant workers. Moreover, the composition of crude oil is geographic region-specific. The degree to which the toxicity of vapors derived from crude oils extracted at different gas/oil plays would give rise to similar biological effects is unknown but is important for worker safety.
